# Humanised Environmental Enrichment: Spatial Effects of Cities and Buildings on Adult Hippocampal Neurogenesis in Humans

**DOI:** 10.3390/ijms27114779

**Published:** 2026-05-26

**Authors:** Mohamed Hesham Khalil

**Affiliations:** Department of Architecture, University of Cambridge, Cambridge CB2 1PX, UK; mhmhk2@cam.ac.uk

**Keywords:** adult neurogenesis, hippocampus, humans, mice, rodents, brain-derived neurotrophic factor, depression, Alzheimer’s disease, cognitive impairment, spatial complexity, physical activity

## Abstract

Adult hippocampal neurogenesis persists throughout the human lifespan, yet declines in Alzheimer’s disease and major depression, associated in part with reduced brain-derived neurotrophic factor (BDNF) levels. For rodents, environmental enrichment, dichotomised primarily as physical activity and spatial complexity, robustly promotes adult hippocampal neurogenesis, but no framework has translated these findings to human environments. This review is the first to synthesise evidence across the full translational pathway, arguing that spatial complexity and physically active navigation in neighbourhoods and buildings constitute a humanised form of environmental enrichment. It proposes that standard indoor environments may represent a functionally impoverished condition for the human hippocampus, paralleling standard laboratory caging. An applied model is presented, mapping built environment features onto the neurobiological mechanisms regulating adult hippocampal neurogenesis, with BDNF as the central translatable biomarker linking environmental exposures to neurogenic outcomes. A methodological roadmap for future empirical validation is also outlined. This framework repositions the built environment as a modifiable determinant of adult hippocampal neurogenesis in humans, with implications for mitigating the risk of depression, cognitive impairment, and Alzheimer’s disease.

## 1. Introduction

Converging evidence from immunohistochemistry and carbon-14 birth-dating, synthesised in a systematic review of primate studies, strongly supports the persistence of adult hippocampal neurogenesis, including in humans [1], yet the understanding of environmental enrichment mechanisms supporting adult hippocampal neurogenesis remains exclusive to rodents, to date. After it was estimated that 700 new neurons are added to the hippocampus per day within a renewing pool comprising approximately 35% of dentate gyrus neurons [2], and that adult hippocampal neurogenesis persists into at least the tenth decade of human life [3,4], the latest evidence demonstrated a decline in markers of cognitive decline [5,6], mediated by changes in brain-derived neurotrophic factor (*BDNF*) gene expression [7], and in neuropsychiatric conditions such as major depression [8]. This growing evidence provides an opportunity to develop a humanised understanding of environmental enrichment for adult hippocampal neurogenesis by examining the mechanisms commonly reported in both humans and rodents that affect biological, cognitive, and affective outcomes associated with adult hippocampal neurogenesis. To address this timely gap and advance the ongoing research on adult hippocampal neurogenesis in humans through providing a humanised understanding of environmental enrichment, this review performs the following: First, this review examines the current state of evidence for adult hippocampal neurogenesis in humans, identifying the markers associated with cognitive decline, psychiatric disorders, and the role played by BDNF peripherally and in the hippocampus. Second, it evaluates the translatability of rodent environmental enrichment to human environments by examining the mechanisms underlying sustained or elevated levels of the identified markers in humans with Alzheimer’s disease (AD) or major depressive disorder (MDD) [5,6,7,8]. This approach is supported by an acknowledgement of convergent biological mechanisms across species, despite human-specific divergent gene expression [9], and by accounting for the commonly shared BDNF-TrkB-dependent trigger of downstream Akt phosphorylation in humans and rodents that drives both proliferation and differentiation [10]. Third, it outlines a methodological roadmap for future interdisciplinary or transdisciplinary research on humans, while highlighting the limitations of each approach based on the latest available evidence.

## 2. Adult Hippocampal Neurogenesis in Humans

The quantitative dynamics of adult hippocampal neurogenesis were first characterised by Spalding et al. [2], who measured ^14^C concentration in genomic DNA from hippocampal neurons (*n* = 55) and non-neuronal cells (*n* = 65) in subjects aged 19–92 years, and found that a large subpopulation constituting 35% (95% CI [12%–63%]) of hippocampal neurons is subject to exchange, corresponding to the vast majority of dentate gyrus (DG) neurons. The remaining ~65% of hippocampal neurons form a nonrenewing fraction that is not replaced once lost. Within this renewing fraction, the median annual turnover rate was 1.75%, corresponding to an estimated ~700 new neurons added to the hippocampus per day (~0.004% of DG neurons). This turnover represents a steady-state process within the renewing pool, where neuronal birth and death are balanced. However, the total number of DG neurons still declines with age because neurons in the nonrenewing fraction die at a slow rate (~0.35% per year) without replacement, resulting in a net loss over time. Separately, the rate of renewal within the renewing fraction also showed a statistically significant but relatively small decline with ageing (*r* = −0.31, *p* = 0.03), with no significant difference by biological gender, representing only an approximate 4-fold decrease across the entire adult lifespan. Adult-born neurons in the renewing fraction had a half-life of 7.1 years, approximately 10× shorter than neurons in the non-renewing fraction, indicating preferential loss of adult-born neurons.

Whether this neurogenic capacity persists across the full adult lifespan became contentious after several studies reported contradictory findings. Cipriani et al. [11] reported that the density and proliferative capacity of neurogenesis are strongly reduced during childhood until 5 years, while Sorrells et al. [12] reported that the number of proliferating progenitors and young neurons in the dentate gyrus sharply drops during the first year of life, with only a few isolated young neurons observed by 7 and 13 years of age, and none detected in adults between 18 and 77 years of age. In contrast, Boldrini et al. [3] demonstrated that the major stages of adult hippocampal neurogenesis, from proliferation to early neuronal maturation, remain intact in ageing humans, with stable cell counts observed across the DG into the eighth decade of life. In 28 healthy individuals (17 males, 11 females; aged 14 to 79 years), with no neuropsychiatric disease or psychotropic medication use and low recent life stress, quiescent neural progenitors (Sox2+) numbered approximately 1000 per DG region and declined selectively in the anterior–mid DG with ageing (Table 1), while nestin+ and Sox2/nestin+ intermediate neural progenitors (INPs), also in the range of approximately 1000 per region (~3000 total per DG), remained stable across all ages (Table 1). Ki-67+ proliferating cells were approximately 10,000 per DG region (~30,000 total per DG) and did not decline between 14 and 79 years of age (Table 1). Crucially, doublecortin-positive (DCX+) and DCX/PSA-NCAM+ immature neurons were preserved at a few thousand per DG region, yielding an estimated 10,000–15,000 new type III INPs or immature neurons per subject at the time of death. The number of NeuN+ mature granule neurons and Nissl+ glial cells remained stable throughout ageing (Table 1), and overall DG volume was preserved across the 65-year age span. However, angiogenesis declined significantly in the anterior-mid DG with ageing, and these decreases correlated with fewer PSA-NCAM+ cells (Table 1), suggesting diminished neuroplasticity. These findings indicate that the dentate gyrus retains substantial neurogenic potential throughout adulthood, although reduced angiogenesis and a smaller quiescent progenitor pool may contribute to age-related declines in cognitive-emotional resilience.

Whereas Spalding et al. [2] detected a modest decline in overall neuronal turnover and a net loss of DG neurons but could not resolve which neurogenic stage was affected, Boldrini et al. [3] localised age-related changes to the upstream quiescent stem cell pool (Sox2+; anterior–mid DG only), angiogenesis, and neuroplasticity markers (PSA-NCAM+), with proliferating progenitors, immature neurons, mature granule neurons, and DG volume all remaining stable.

Further confirmation came from Moreno-Jiménez et al. [5], who identified thousands of DCX+ immature neurons in the DG of 13 neurologically healthy subjects (aged 43 to 87 years), using tightly controlled tissue processing methods and validating specificity by the absence of DCX signal in non-neurogenic regions. Even into the ninth decade, a significant population of immature neurons persisted, exhibiting a wide range of maturation states consistent with a prolonged neurogenic process. The number of DCX+ cells decreased moderately with age (*r* = −0.5842, *p* = 0.036) (Table 1). Tobin et al. [6] corroborated these findings in a cohort of 18 participants with a mean age of 90.6 years, detecting neural progenitor cells marked by Nestin and Sox2, as well as DCX+ neuroblasts and immature neurons. Estimated mean counts were approximately 127,342 ± 28,864 DCX+ cells and 3054 ± 1149 DCX+PCNA+ proliferating neuroblasts per dentate gyrus. The DCX+ range of 3000 to 30,000 cells per dentate gyrus was similar to that reported by Boldrini et al. [3], yet lower than the 5000 to 45,000 cells per cubic millimetre observed by Moreno-Jiménez et al. [5]. The number of DCX+PCNA+ cells was significantly reduced in Mild Cognitive Impairment (MCI) compared to cognitively normal individuals (*p* = 0.038), and higher numbers of these proliferating neuroblasts correlated with better global cognitive scores (*p* = 0.046) (Table 1).

Beyond this inter-individual variation, adult hippocampal neurogenesis differs markedly between healthy and cognitively impaired brains. Tobin et al. [6] reported that individuals with MCI had significantly fewer DCX^+^/PCNA^+^ cells than cognitively normal controls (*p* = 0.038), and, when MCI and AD participants were combined, the trend persisted, though it did not reach significance (*p* = 0.068). Moreno-Jiménez et al. [5] reported that AD samples showed a profound and progressive reduction in DCX^+^ cells, beginning in early disease stages and worsening with severity (Table 1). The number of DCX^+^ cells in neurologically healthy individuals of any age was consistently higher than in AD patients, regardless of age. Beyond the decline in cell numbers, impaired maturation was also evident, with reductions in PSA-NCAM expression beginning at Braak stage III (*p* < 0.0001), followed by decreases in NeuN at subsequent stages (*p* = 0.0215) (Table 1).

With the growing evidence, the debate no longer centres on the persistence of adult hippocampal neurogenesis in humans but on what drives variability among healthy subjects and between healthy subjects and AD/MCI patients. Moreno-Jiménez et al. [4] noted that differences in tissue processing or histologic methodologies can limit the detection of several markers of adult hippocampal neurogenesis to the point of rendering them undetectable, a view supported by growing evidence of the persistence of adult hippocampal neurogenesis in humans. Yet, while the same reason may explain some of the aforementioned variability, not all studies controlled for confounding environmental-enrichment variables that may have influenced the outcome.

Understanding the relationship between environmental enrichment and adult hippocampal neurogenesis has become more feasible following the identification of biomarkers associated with cognitive decline and psychiatric disorders.

After understanding that adult hippocampal neurogenesis drops in patients with AD [5,6], Disouky et al. [7] have extended these observations in a study across five cognitive groups: young adults (YA), healthy agers (HA), preclinical intermediate pathology (PCI), AD, and SuperAgers (SA). They confirmed that the average number of neuroblasts and immature neurons was significantly reduced in the AD group compared with both the HA and YA cohorts, and that immature neurons were also significantly reduced in AD relative to PCI. Intriguingly, neural stem cell numbers were significantly higher in PCI and AD than in HA. A key advance of this study was demonstrating that epigenetic remodelling represents a more robust molecular signature of cognitive decline. While SA exhibited a significant increase in immature neurons compared with the AD group (approximately 2.5-fold increase relative to other cohorts, even after outlier exclusion), this change was accompanied by upregulation of the *BDNF* gene (Table 1).

Reduced adult hippocampal neurogenesis is not limited to cognitive decline but extends to psychiatric disorders. Boldrini et al. [13] provided early evidence that hippocampal neurogenesis and angiogenesis are coupled in the human DG and are modulated by antidepressant treatment in major depressive disorder (MDD). Using nestin as a marker of neural progenitor cells (NPCs), they found that treated MDD patients had significantly more NPCs than both untreated MDD patients (*p* = 0.008) and non-psychiatric controls (*p* = 0.002) across the DG. Critically, untreated MDD patients did not differ from controls in NPC number (Table 1), suggesting that the neurogenic deficit in depression may be subtle or confined to later maturation stages. Age negatively correlated with NPC number and capillary bifurcations, while no sex effect was detected. More recently, Márquez-Valadez et al. [8] confirmed and expanded that human adult hippocampal neurogenesis is profoundly shaped by neuropsychiatric disorders, including MDD, schizophrenia (SCH), and bipolar disorder (BD). The density and proliferative capacity of neural stem cells (NSCs) and neuroblasts were significantly reduced (*p* < 0.01–0.001) in those conditions (Table 1). However, the maturation profile differed by disorder: in MDD, DCX^+^ and PSA-NCAM^+^ immature dentate granule cell (DGC) densities remained unchanged (Table 1), suggesting selective vulnerability of early neurogenic stages, whereas in SCH and BD, these densities were paradoxically increased (Table 1), potentially reflecting compensatory survival responses or impaired neuronal maturation progression. Demographics (age and biological sex) as well as lifestyle factors (alcohol and drug consumption) further modulate adult hippocampal neurogenesis in both psychiatric and healthy populations, underscoring the need for control of these variables in future research. The two MDD studies show partial consistency, as both identify age-related declines in hippocampal neurogenesis and vascular complexity, and both implicate the early proliferative stages as key targets of disruption, but they diverge on a critical point. Boldrini et al. [13] found no difference in NPC number between untreated MDD and controls, while Márquez-Valadez et al. [8] detected reduced NSC density and proliferative capacity in MDD.

In Table 1, columns represent markers spanning the three phases of neurogenesis: proliferation (Sox2, Nestin, Ki-67/PCNA), differentiation (DCX), and survival (PSA-NCAM, NeuN), although some markers may span more than one phase. Rows represent six clinical conditions. Healthy ageing preserves most markers through the eighth decade, with only modest DCX decline reported across studies. AD shows a paradoxical increase in Sox2+ neural stem cells, likely reflecting a failed compensatory attempt, followed by a progressive collapse beginning at the differentiation stage. MCI shows selective loss of proliferating neuroblasts. In MDD, early proliferative stages are disrupted while maturation-stage markers remain intact, contrasting with the pattern in AD. SCH and BD show paradoxical increases in DCX and PSA-NCAM, possibly reflecting compensatory responses.

It can be argued that BDNF may also explain the association between adult hippocampal neurogenesis and MDD in humans. In a review by Cattaneo et al. [14], both peripheral *BDNF* gene expression and blood protein levels are relevant to understanding depression and antidepressant response in humans. Serum and plasma BDNF protein levels are consistently reduced in drug-free depressed patients and tend to normalise following effective treatment, suggesting that low BDNF protein serves as a state marker of depression rather than a stable trait. *BDNF* mRNA is similarly reduced in depressed patients and correlates with baseline and treatment serum protein levels, indicating that changes in blood protein levels may partly reflect altered synthesis in white blood cells rather than platelet release alone. Importantly, *BDNF* mRNA expression in peripheral blood may more accurately reflect the central mechanisms of *BDNF* physiology than protein levels do, given the overlap between blood cell and brain mRNA profiles.

With a clear understanding of how adult hippocampal neurogenesis in humans may be explained by cognitive decline, MDD, and peripheral serum BDNF, in the absence of markers of gene expression. It is possible that environmental enrichment can be understood from rodent studies based on their association with the markers in question and from human studies based on the biological, cognitive, and affective markers in question, and if they happen to be the same environmental enrichment mechanisms, it can become possible to have a preliminary understanding of environmental enrichment for humans.

The evidence reviewed in this section falls into three categories of certainty. First, the decline of adult hippocampal neurogenesis markers, including DCX+, PSA-NCAM+, and NeuN+, in AD, and of DCX+ specifically in MCI, and evidence of disruption to early proliferative stages in MDD, though findings across studies are not fully consistent, is directly supported by postmortem immunohistochemical studies in humans [5,6,7,8,13]. Hippocampal *BDNF* gene expression has also been directly shown to mediate the relationship between neurogenesis markers and cognitive resilience in postmortem tissue [7]. The role of BDNF differs between these conditions: in AD, hippocampal *BDNF* gene expression directly mediates the relationship between neurogenesis markers and cognitive resilience [7], whereas in MDD, peripheral BDNF protein serves as a state marker of depression that normalises following effective treatment rather than reflecting hippocampal neurogenesis directly [14]. Second, the association between peripheral BDNF levels and depression state is based on correlational human evidence and does not establish a direct causal link to hippocampal neurogenesis [14]. Third, whether inter-individual variability in neurogenesis markers is driven by environmental factors, including the built environment, remains an untested hypothesis that motivates the translational framework developed in the following section.

## 3. Environmental Enrichment Mechanisms and Adult Hippocampal Neurogenesis in Rodents

Cross-species comparisons reveal several notable similarities and differences to consider in the translatability of environmental enrichment from rodents to humans. Maturation time is longer in primates than in rodents [15]. Additionally, whether the differences in adult hippocampal neurogenesis between humans and rodents discussed by Spalding et al. [2] extend to the molecular level is less straightforward. Recently, Zhou et al. [9] found that immature dentate granule cells across humans, macaques, pigs, and mice converge on shared biological processes, including neurogenesis, neuronal development, and synaptic plasticity, but use divergent sets of genes to achieve these processes. Only 9 out of 541 human Immature granule cells (imGC)-enriched genes (1.6%) were shared across all four species, and even between humans and macaques, the overlap was just 6.8%. This molecular divergence warranted further exploration of what is commonly shared between rodents and humans. However, the BDNF-TrkB signalling pathway shows notable conservation across species, where Charou et al. [10] demonstrated that a novel TrkB agonist increased neural stem cell proliferation and protected against amyloid-β toxicity in both mouse primary hippocampal/cortical neural stem cells and human neural progenitor cells from healthy and AD (*APOE4*) donors. BDNF here promotes adult hippocampal neurogenesis by binding and activating TrkB, triggering downstream Akt phosphorylation and promoting cell proliferation and differentiation. Yet, species differences emerge again. The compound regulated nearly 8-fold more genes in human cells than *BDNF* alone did, suggesting broader signalling engagement in human neural progenitors. Collectively, these findings validate BDNF-TrkB as a translational target while underscoring that the downstream molecular response in human cells is likely far richer and more complex than rodent models suggest. Rodent studies can identify promising pathways, but they will systematically underestimate the breadth of molecular engagement in humans. Therefore, developing an understanding of environmental enrichment for humans can advance research on adult hippocampal neurogenesis and the human-specific molecular mechanisms underlying it.

The foundational studies on environmental enrichment and adult hippocampal neurogenesis established independent effects of spatial complexity and running wheels. While the earliest study demonstrating the effect of environmental enrichment on adult hippocampal neurogenesis in mice used both mechanisms without separation [16], Van Praag et al. [17] dissected this further by assigning adult mice to five conditions for ~40 days: water-maze learning, yoked swimming, voluntary running wheel, enriched environment, and standard housing. Water-maze training and forced swimming had no effect on cell proliferation or neurogenesis (Table 2). Both voluntary running and enrichment roughly doubled the total number of surviving newborn cells compared to controls (3791 and 3282, respectively, compared to 1880), but through different mechanisms. Only runners showed increased cell proliferation (6773 vs ~4000 in other groups) (Table 2), whereas enrichment dramatically increased the survival rate of normally born cells (42–46% survival in most groups vs 85% in enriched, 56% in runners). Running nearly doubled surviving cells by massively increasing cell birth (6773 vs ~3867 in enriched) despite a relatively high attrition rate (~2982 cells lost), whereas enrichment achieved a comparable outcome by retaining almost all of its normally produced cells (~585 lost). These two pathways, proliferation via physical activity and survival via cognitive enrichment, are fundamentally distinct yet complementary in this context. *Bdnf* gene expression has been reported in enrichment models containing running wheels [18,19,20,21]. Yet, independent of the effects of running wheels or physical activity, research continues to show that cognitive enrichment can effectively promote neurogenesis in rodents. Funabashi et al. [22] found that Ki-67 showed no significant difference between environmental enrichment and standard-housed mice, yet DCX was significantly elevated in environmental enrichment mice (*p* < 0.01) (Table 2). Similarly, Birch and Kelly [23] found that Ki67 mRNA in the DG declined with age regardless of housing condition, indicating that environmental enrichment without exercise did not rescue proliferative capacity, while BrdU+ cells were significantly preserved in aged environmental enrichment rats compared with aged standard-housed rats. Taken together, the evidence supports the early findings by Van Praag et al. [17] suggesting that physical activity and environmental enrichment are independent but complementary. It will still be irrelevant to translate the effects of tunnels, toys, and running wheels into a human context, which leads to two critical questions.

First, whether physical activity, which can be sufficient for adult hippocampal neurogenesis, can be sufficiently achieved through the increase in an indoor environment footprint or an outdoor environment range. In an indoor environment, Funabashi et al. [22] demonstrated that progressively increasing cage size and introducing spatial complexity through varied objects, both without a running wheel, did not increase physical activity in mice (Table 2), as measured by body-implanted actinometers. Despite this, the enriched mice showed significantly higher densities of DCX+ immature neurons, while Ki67+ cell proliferation was unchanged, indicating that environmental complexity promoted neuronal survival or differentiation rather than new cell birth, consistent with Van Praag et al. [17], but suggesting that enlarging housing is not a substitute for running wheels. For an outdoor environment, by contrast, Sinks et al. [24] showed that free-ranging male meadow voles with larger home ranges, spanning hundreds to thousands of square metres of natural habitat, displayed significantly higher densities of proliferating cells (Ki67+) in the DG, yet showed no correlation between home range size and DCX+ cell density. The vastly greater spatial scale and locomotor demands of natural ranging behaviour appear sufficient to drive cell proliferation in a way that an indoor environment cannot. These findings suggest that, for humans, outdoor environments may support cell proliferation when opportunities for voluntary, but not forced, physical activity are present, such as walking, whereas achieving sufficient physical activity levels may be more difficult in indoor environments.

Second, cognitive enrichment through complex layouts increases markers of survival, despite and likely independently of running wheels. In the Hamlet complex maze [25], training increased not only proliferation markers (BrdU at 24 h, Ki67) but also survival and maturation markers: BrdU+ cells persisted at two weeks post-injection, and DCX+ immature neurons (Table 2) showed significantly greater dendritic branching and total dendritic length. The finding that low-dose scopolamine (0.5 mg/kg) blocked training-induced increases in both Ki67 and DCX without impairing topographic memory performance is particularly informative, as it suggests that cognitive processing, rather than physical activity, is a driver of the neurogenic response or at least part of it, further supported by further analysis that confirmed that the hippocampus–subiculum–parahippocampal gyrus axis was sustainably recruited during maze exploration. Similarly, in the Marlau cage [26], BrdU+ cells measured 14 days post-injection nearly doubled in enriched rats, with over 90% co-labelling with NeuN (Table 2). Taken together, despite the limitation of running wheels, the findings suggest that complex layouts can support survival rather than proliferation.

As presented in Table 2, voluntary physical activity, primarily via running wheels, selectively increases cell proliferation, reflected in elevated Ki-67+ and BrdU+ counts at 24 hours, without substantially improving cell survival rates. Cognitively stimulating environments, including novel objects, maze navigation, and complex layouts, selectively enhance the survival and maturation of newly born cells, reflected in elevated BrdU+ counts at two weeks and greater DCX+ dendritic complexity, without necessarily increasing proliferation. Forcing swimming also confers no neurogenic benefits, likely due to the elevated stress that can impair neurogenesis. These two pathways are dissociable yet complementary, and their distinction motivates the separate treatment of physical activity opportunities and spatial complexity as independent environmental enrichment determinants in human-built environments.

**Table 2:** Environmental enrichment mechanisms and their effect on adult hippocampal neurogenesis in rodents.

Rodent Models				
Environmental Enrichment		Neurogenesis Markers		BDNF
Mechanism	Application	Proliferation Ki-67^+^/BrdU^+^ (24 h)	Survival/Maturation BrdU^+^ (2 wk)/DCX^+^	
Voluntary physical activity	Running wheel [17]; large home range [24]	↑ (6773 vs. ~4000) [17]; ↑ Ki-67^+^ [24]	→ [17]; → DCX^+^ [24]	↑ hippocampal BDNF [18,19,20,21]
	Enlarged housing without running wheel [22]; Forced swimming [17]	→ [17,22]	→ [17]	—
Spatial complexity and navigation	Novel objects [17]; enlarged housing + complexity [22]; lifelong enrichment without exercise [23]; Marlau cage [26]; Hamlet complex maze [25]	→ [17,22,23]; ↑ Ki-67^+^, BrdU^+^ (24 h) (running wheel present) [25]	↑ (85% vs. 42–46%; ↑ DCX^+^) [17]; ↑ DCX^+^ [22]; ↑ BrdU^+^ [23]; ↑ BrdU^+^ (×2; >90% NeuN) [26]; ↑ BrdU^+^ (2 wk), ↑ DCX^+^ dendritic complexity, and ↑ hippocampal circuit activity [25]	—
	Morris water maze [17]	→ [17]	→ [17]	—

↑ indicates an increase; → indicates no change.

## 4. Humanised Environmental Enrichment: Potential Effects of Cities and Buildings on Neurogenic Markers

MDD in humans is associated with reduced proliferation and differentiation [8,13], likely indicating a physical activity-associated insufficiency rather than a lack of cognitive enrichment affecting survival, and this argument can be supported by the role played by BDNF in serum and plasma acting as a state marker of depression, and *BDNF* gene expression as a reflection of the central mechanism of *BDNF* gene expression [14]. In human environments, at both outdoor and indoor scales, it is possible to explain the relationship between environmentally mediated physical activity opportunities and the lack of such opportunities using evidence from serum or plasma BDNF and depression as outcomes. First, based on the finding by Sinks et al. [24] that larger home ranges spanning hundreds to thousands of square metres of natural habitat increased proliferation, and where running wheels increase *Bdnf* gene expression while promoting neurogenesis [18,19,20,21], it can be hypothesised that greater walking opportunities outdoors can have similar benefits, relying on serum BDNF as a biomarker. For instance, a 30-minute walk at moderate intensity in an indoor-environment temperature significantly elevated serum BDNF for pregnant and non-pregnant women (*p* ≤ 0.001 for non-pregnant; *p* = 0.025 for pregnant) [27]. A 180-minute walk of moderate intensity increased BDNF in men who walked in a hot environment (32 °C), significantly (*p* < 0.05), but not in a temperate environment (16 °C) [28]. None of a 3.5 h moderate intensity 18-hole round of golf walking, a 3.5 h moderate-to-vigorous 6km Nordic walk, or regular walking increased BDNF immediately post-walk, while the Nordic walking showed a delayed increase in BDNF (*p* = 0.046) [29], which can be due to the counteraction of prolonged vigorous activity [30]. A 5 h 18-hole round of golf walking, in contrast, elevated serum BDNF concentrations by 1.2 ± 0.3-fold immediately post exercise [31]. The 18-hole golf walk is the closest example to the larger home ranges studied by Sinks et al. [24], suggesting that environmental opportunities for physical activity can elevate BDNF levels in humans. Second, based on the aforementioned evidence, it is doubtful that walking indoors would increase BDNF in humans; even though the evidence is lacking here, complementary evidence suggests that smaller footprints are more likely to elevate depression in humans. For instance, smaller apartments (<60 m^2^) increased the odds ratio (OR) of depression (OR = 1.31) [32], while living in larger homes (7+ rooms) had significantly lower depression rates (OR = 0.76) compared to those in medium-sized homes, while those in single-room apartments had higher rates (OR = 1.55) [33]. While this remains unclear, it is hypothesised that stair use in larger homes can increase BDNF levels in humans [34]. Collectively, walking increases BDNF in humans, and Funabashi et al. [22] suggested that increasing footprint is unlikely to increase physical activity in rodents; it is unlikely that enlarging the footprint in indoor environments can increase BDNF, but it is very likely that it increases the risk of depression. In this context, adult hippocampal neurogenesis in humans can become dependent on the average of both opportunities.

While homebodies who rarely leave their home are at higher risk of cognitive impairment [35], human outdoor environments with higher layout complexity are associated with lower AD risk and sustained or increased hippocampal volume [36,37,38], which suggests the possibility that mazes that support adult hippocampal neurogenesis in rodents [25,26], given that they increased survival and not proliferation in rodents and since AD in humans is associated with a drop in DCX and PSA-NCAM but not in Sox2 [5,6]. Lövdén et al. [36] reported that, compared with a yoked control group who walked without a navigational task, the complex-landscape navigation training group showed stable hippocampal volumes across both the training phase and a 4-month follow-up period, whilst controls exhibited volume decrements consistent with normal age-related decline. Cerin et al. [37] reported that, longitudinally, *APOE ε4* carriers in more walkable areas showed significantly slower hippocampal decline and reduced amyloid accumulation over 18 months, with physical activity explaining only a small proportion of these associations, suggesting that the navigational and cognitive demands of complex environments themselves drive hippocampal preservation. Shin et al. [38] extended this understanding of spatial complexity to the real-world residential environment, using structural equation modelling in a sample of 660 older adults drawn from the National Alzheimer’s Coordinating Centre dataset spanning the full Alzheimer’s spectrum. Individuals residing in zip-code areas with greater geospatial complexity, quantified by road network entropy and point-of-interest density, showed selectively larger allocentric navigation-related brain volumes and reduced AD risk.

These findings may help hypothesise that some of the observed variability in adult hippocampal neurogenesis may be attributed to geospatial complexity variability, but without assuming causality at this stage. It is notable that the subjects in Tobin et al. [6] were drawn from longitudinal community-based studies centred at Rush University Medical Centre in Chicago, Illinois, a location that the geospatial environmental complexity index in Shin et al. [38] classifies as having moderate-to-high spatial complexity and lower AD risk. While this observation is correlational and cannot establish causation, it raises the possibility that environmental enrichment may partly explain why Tobin et al. [6], unlike Moreno-Jiménez et al. [5], detected adult hippocampal neurogenesis even in AD patients.

**Figure 1:**
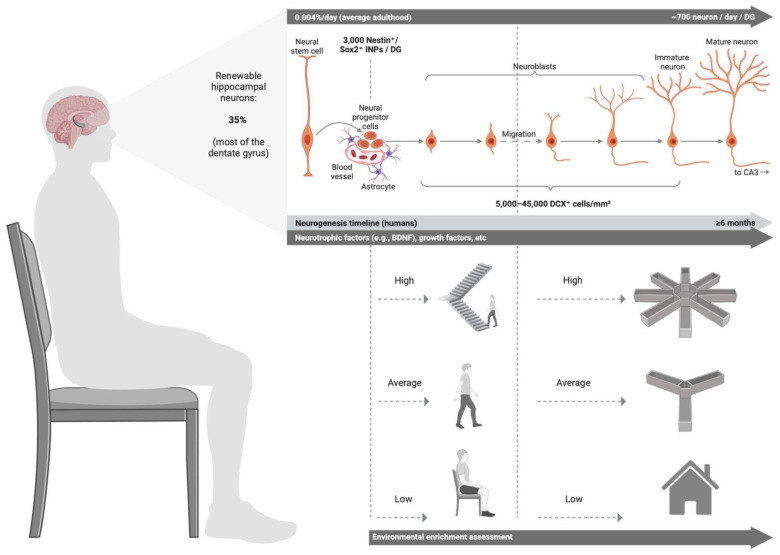
Conceptual model of adult hippocampal neurogenesis in humans and its environmental determinants. The upper panel illustrates the neurogenic process in the human dentate gyrus, from neural stem cells through neural progenitor cells, neuroblasts, and immature neurons to mature granule cells that arguably integrate into the cornu ammonis 3 (CA3) circuit in the human brain, as in rodents. Approximately 35% of dentate gyrus neurons are renewable, with ~700 new neurons added per hippocampus per day at a turnover rate of 0.004% per day during average adulthood, and DCX+ neuroblast densities ranging from 5000 to 45,000 cells/mm^3^ across studies. The full maturation of a new neuron in humans is estimated to take at least six months and depends on sustained neurotrophic support, including BDNF. The lower panel proposes an environmental enrichment assessment framework for humans along two independent but complementary dimensions: spatially mediated physical activity, ranging from sedentary indoor living to stair use and outdoor walking or running; and spatial complexity of the built environment, ranging from standard single-family housing to complex multi-path layouts. Together, these dimensions can determine the degree to which the built environment may support adult hippocampal neurogenesis in humans. Created in BioRender. Khalil, M. (2026) https://BioRender.com/38x978h (accessed on 20 May 2026).

The evidence reviewed here, despite the limitations associated with translatability, charts a consistent trajectory from rodent to human environments. Although the hypothesis that standard indoor living constitutes a functionally impoverished condition similar to standard housing conditions for rodents, outdoor physical activity and opportunities for spatial complexity are worth exploring in future research. This review synthesises the evidence (Table 3) to propose an applied model of environmental enrichment for humans (Figure 1). The model positions time spent indoors relative to time spent outdoors and considers the effects of spatial complexity and environmentally mediated physical activity as two complementary outdoor determinants, which may help future research explore their association with adult hippocampal neurogenesis in humans.

Before proceeding to future directions, it is important to distinguish what the evidence reviewed in this section does and does not establish. The effects of voluntary physical activity and spatial complexity on hippocampal neurogenesis markers are directly established in rodents [17,22,23,24,25,26]. The associations between walking and peripheral BDNF, and between neighbourhood complexity or housing typology and hippocampal volume, cognitive function, or depression risk, are directly observed in humans but remain correlational [27,28,29,30,31,32,33,35,36,37,38]. The mapping of these human associations onto the rodent enrichment mechanisms constitutes cross-species inference rather than established equivalence. Testing whether outdoor walking, spatial navigation, and residential complexity jointly constitute a humanised form of environmental enrichment capable of modulating adult hippocampal neurogenesis is the central question this framework is designed to address.

## 5. Future Directions

The previous sections explained that environmental enrichment sustains adult hippocampal neurogenesis in rodents through two primary mechanisms: voluntary physical activity and environmental complexity, suggesting that these mechanisms also underlie the biomarker, cognitive, and affective aspects of adult hippocampal neurogenesis in humans. Despite the limitations associated with translational research, the current review identifies the environmental enrichment mechanisms that can be carried forward for future research on humans, specifically where indoor environments, specifically housing, are classified as impoverished for humans based on its increase in risk for depression and cognitive impairment [32,33], which are associated with a drop in adult hippocampal neurogenesis in humans [5,6,7,8]. Specifically, geospatial environmental complexity is associated with lower AD risk in humans [37,38], while ample outdoor environments can increase serum BDNF after prolonged walking [31] or during shorter durations at higher intensities, or under heat exposure, to compensate for low walking intensity [27,28]. This review does not assume causality but provides the timely theoretical foundation necessary for future research, which may involve interdisciplinary or transdisciplinary efforts that rely on neurogenesis markers, biomarkers, and cognitive or affective outcomes.

However, the framework presented in this review rests on three levels of evidence that should be clearly distinguished before outlining future directions. First, the decline of adult hippocampal neurogenesis markers in AD, MCI, and MDD, and the association of peripheral BDNF with depression state and cognitive resilience, are directly supported by postmortem and clinical evidence in humans [3,5,6,7,8,13,14]. Second, the mapping of voluntary physical activity and spatial complexity onto hippocampal neurogenesis mechanisms is based on cross-species inference from rodent environmental enrichment studies [17,22,23,24,25,26]. Within the human literature, this inference is supported by converging but indirect evidence: peripheral serum BDNF increases following prolonged walking [27,28,31], suggesting that outdoor physical activity can engage the same BDNF-dependent pathway identified in rodents; walkable neighbourhoods with greater geospatial complexity are associated with larger hippocampal volume and reduced AD risk [36,37,38], paralleling the survival-promoting effects of complex environments in rodents [22,25,26]; and smaller or spatially restricted indoor environments are associated with increased depression risk [32,33], consistent with the impoverished housing model. However, none of these human associations has been tested against direct neurogenesis markers, and causality has not been established. Third, the proposition that the built environment, particularly indoor spatial impoverishment and outdoor navigational complexity, directly modulates adult hippocampal neurogenesis in humans, and thereby influences depression and AD risk, remains an untested hypothesis and constitutes the central question this framework is designed to motivate.

A cascade of methods can be used in future research, each coming with limitations. First, for peripheral measures, BDNF concentrations increase immediately after physical activity. While serum BDNF is suggested to predict state depression, unlike *BDNF* mRNA [14], it remains a non-invasive method, and salivary BDNF is even more non-invasive. A 15-minute high-intensity interval training session can increase salivary BDNF by 25% [40], but it remains unreliable for lower-intensity activities. Several commercial Enzyme-linked immunosorbent assay (ELISA) kits are not supported to test BDNF in saliva [41]. Peripheral measures have limitations and should be interpreted cautiously, as they are unlikely to directly reflect changes in the central nervous system. Second, peripherally released BDNF may potentially cross the blood–brain barrier (BBB) to exert central effects. Pan et al. [42] demonstrated that BDNF in mouse serum remained stable in blood for up to 60 minutes and that it rapidly entered the brain via a high-capacity, saturable transport system. Poduslo & Curran [43], using rats, found that the permeability coefficient–surface area product for BDNF across the BBB was 11–16 times higher than that of nerve growth factor across six brain regions. Despite an extremely short plasma half-life of only 0.92 minutes, intact BDNF protein was detected in multiple brain regions after 60 minutes of uptake. Whether BDNF crosses the BBB in humans as well remains questionable. Third, despite divergent molecular mechanisms across species, they share biological processes [9], specifically BDNF-TrkB binding, which triggers downstream Akt phosphorylation and drives both proliferation and differentiation [10], necessitating that BDNF remains an appropriate biomarker despite its limitations. Furthermore, BDNF is associated with an antidepressant effect in humans [14], while its peripheral levels are inversely associated with depression [14], and hippocampal *BDNF* gene expression mediates the relationship between adult hippocampal neurogenesis and cognitive resilience in older adults compared to AD patients [7]. Collectively, adopting a BDNF-centric approach to studying the relationship between the proposed environmental enrichment mechanisms in the human environment, yet it does not replace the need to associate the mechanisms with adult hippocampal neurogenesis markers in postmortem samples whenever feasible. The mapped conceptual association between environmental enrichment in human environments and adult hippocampal neurogenesis remains important for therapeutic and preventive cognitive and affective risk outcomes [44,45], which can be impaired by insufficient environmental enrichment.

Peripheral BDNF is used throughout this review as the primary translational biomarker linking built environment exposures to neurogenic outcomes, but its limitations as a proxy for central neurogenesis require systematic acknowledgement. First, regarding sources, peripheral BDNF is mainly stored in platelets, accounting for approximately 99% of circulating BDNF, with only a small amount of free BDNF present in plasma [46]. During blood coagulation, platelet activation causes a release of BDNF into serum, meaning serum BDNF largely reflects peripheral platelet dynamics rather than neural production directly [46]. White blood cells contribute an additional source through independent *BDNF* transcription [14]. Second, plasma BDNF, obtained using anticoagulant tubes that prevent platelet activation and BDNF release, provides a less confounded measure; since platelets cannot pass the blood–brain barrier, free plasma BDNF may more closely reflect circulating BDNF relevant to brain function than serum BDNF does [46]. Third, *BDNF* mRNA expression in peripheral blood may more accurately reflect central mechanisms of *BDNF* than protein levels, given the overlap between blood cell and brain mRNA profiles, while protein levels remain more susceptible to platelet contamination and peripheral synthesis [14]. Fourth, the walking and outdoor activity studies cited in this review [27,28,29,30,31] document acute post-exercise elevations in peripheral BDNF. For instance, light cycling increases both serum and plasma BDNF, with serum increases primarily mediated by elevated platelet count, while higher-intensity cycling increases both plasma and serum BDNF as well as the BDNF-per-platelet ratio four to five times more than light exercise, indicating that increasing exercise intensity is required to additionally liberate free BDNF beyond platelet-mediated responses [47]. While informative, these acute spikes may not reflect lasting neurogenic changes; long-term adaptations in resting BDNF levels following sustained exposure to enriched built environments are the more biologically relevant metric for neurogenesis. However, these remain substantially understudied in the context of residential and neighbourhood environments. Fifth, salivary BDNF offers a minimally invasive alternative and can increase by 25% following high-intensity interval training [40], but remains unreliable for lower-intensity activities [41]. Sixth, whether peripheral BDNF crosses the blood–brain barrier to exert central effects in humans remains uncertain. Animal studies suggest BDNF can enter the brain via a high-capacity, saturable transport system [42] with a permeability coefficient substantially higher than that of other neurotrophins [43], but the extent to which this occurs in humans under physiological conditions is unknown. Taken together, peripheral BDNF remains the only non-invasive translational biomarker available at scale for human field studies, justifying its continued use as a proxy with acknowledged constraints. Future studies should prioritise *BDNF* mRNA over protein levels where feasible, account for platelet contamination through plasma rather than serum measurement and distinguish between acute exercise-induced spikes and resting baseline levels when interpreting associations with built environment features.

What constitutes humanised environmental enrichment? Based on the translational framework presented in this review, future research should operationalise human environmental enrichment using explicit and measurable indicators that map onto the rodent enrichment mechanisms identified. For outdoor physical activity, analogous to running wheel use, relevant indicators include average daily walking distance, heart rate, and average daily outdoor time. For outdoor spatial complexity and navigational demand, analogous to maze exposure, road-network entropy and point-of-interest density provide validated quantitative measures [38], which can be coupled with Global Positioning System (GPS)-derived navigation activity and frequency. For indoor spatial complexity, analogous to enriched housing layouts, the Architectural Spatial Complexity Index offers architectural measures of cognitive enrichment potential [39], while frequency and intensity of stair exposure provide an indicator of incidental physical activity within the home [34]. Together, these indicators would allow future longitudinal studies to move beyond laboratory models towards examining the association between built environment exposures and adult hippocampal neurogenesis markers in humans.

Future research should specifically examine the hypothesis that standard indoor environments constitute a functionally impoverished condition for the human hippocampus in populations that spend the majority of their time indoors, including homebodies, retired older adults, and those with limited mobility, for whom the indoor environment may represent the primary or sole source of spatial enrichment. Single-storey apartment residents showed significantly higher depression scores than multi-storey house residents, with psychomotor agitation and retardation as the selectively predicted symptom dimension [48], consistent with the hypothesis of reduced incidental physical activity in the absence of stair infrastructure [34]. Furthermore, single-storey apartments score significantly lower on a validated measure of indoor spatial complexity using the Architectural Spatial Complexity Index (M = 0.23) than multi-storey houses (M = 0.48) [39], a pattern that mirrors the difference between non-enriched (M = 0.15) and enriched rodent housing (M = 0.59) on the same index. Comparative analysis of rodent maze architectures using the A-SCI tool further shows that the simple T-maze scored the lowest among the 16 maze architectures examined (0.14), approaching the range of non-enriched standard housing when assessed separately (M = 0.15 [39]), while pro-neurogenic mazes such as the Hamlet complex maze (0.27) and the Marlau cage (0.35–0.39) scored substantially higher, suggesting that maze complexity, rather than mere maze presence, drives the neurogenic potential [49]. Studying those associations is warranted, taking into account confounding variables such as but not limited to time spent outdoors, socioeconomic status, education, physical health, urban deprivation, air pollution, and social isolation.

Table 4 synthesises the translational mapping developed in this section, summarising the rodent enrichment mechanisms, their neurogenic effects and associated markers, the BDNF bridge linking rodent and human evidence, and the proposed human analogues with their measurable indicators. This mapping is intended to operationalise the framework for future empirical research and to make explicit the cross-species inferences on which the proposed human model rests.

## 6. Conclusions

This review is the first to synthesise evidence across the full translational pathway, from rodent environmental enrichment to the human built environment, proposing that spatially mediated physical activity and spatial complexity in cities and buildings constitute a humanised form of environmental enrichment for adult hippocampal neurogenesis. The two mechanisms are dissociable yet complementary: physical activity, analogous to running wheel use in rodents, can selectively promote proliferation, while spatial complexity, analogous to maze exposure, can selectively promote survival and maturation of newly born neurons. These two pathways map precisely onto the two disease-specific neurogenic deficits identified in postmortem human studies: early proliferative disruption in major depression and maturation-stage collapse in AD and MCI, with BDNF serving as the central translatable biomarker linking environmental exposures to neurogenic outcomes.

Three levels of evidence underpin this framework. The effects of voluntary physical activity and spatial complexity on hippocampal neurogenesis markers are directly established in rodents. The associations between outdoor walking or stair use and peripheral BDNF, and between neighbourhood complexity and hippocampal volume or reduced AD risk, are directly observed in humans but remain correlational. The proposition that the built environment directly modulates adult hippocampal neurogenesis in humans remains an untested hypothesis and constitutes the central question this framework is designed to motivate.

This framework repositions the built environment as a modifiable determinant of hippocampal neurogenesis, with implications for the prevention of cognitive decline, major depression, and AD. Future longitudinal and postmortem research operationalising the measurable indicators proposed here—including outdoor walking opportunities, road-network entropy, street connectivity, building layout complexity, and stair use patterns—will be necessary to test whether humanised environmental enrichment can constitute an informative model for laboratory studies on rodents and a viable non-pharmacological strategy for sustaining adult hippocampal neurogenesis across the human lifespan.

## Figures and Tables

**Table 1:** A synthesis of findings about adult human hippocampal neurogenesis markers across neurogenic stages in healthy ageing, cognitive decline, and psychiatric disorders, synthesised from postmortem immunohistochemical studies.

Subjects	Neurogenesis Markers in Humans					
	Sox2 (Quiescent Progenitors)	Nestin (Intermediate Progenitors)	Ki-67/PCNA (Active Proliferation)	DCX (Neuroblasts/Immature Neurons)	PSA-NCAM (Late Immature Neurons)	NeuN (Mature Neurons)
Healthy ageing	~1000/region ↓ anterior-mid DG only Nestin + Sox2+: 35.4–56.0 cells/mm^3^ at 90 [3,6]	~1000/region ~3000 total/DG → Stable ages 14–79 [3]	~10–30 K total/DG → Stable 14–79 [3]	Preserved 14–79; or ↓~decline with age 10–15 K; 3–30 K/DG; 5–45 K/mm^3^ [3,5,6]	↓~Few K/region Declines with angiogenesis loss [3]	→ Stable counts and DG volume [3]; non-renewing pool: −0.35%/yr [2]
SuperAgers	—	—	—	↑~2.5× vs. others (*BDNF* upregulated) [7]	—	—
Alzheimer’s disease	↑ NSCs increased (compensatory attempt that fails downstream) [7]	—	—	↓ Profound drop healthy > AD at any age + impaired maturation [5]	↓ Drops from Braak stage III maturation failure [5]	↓ Drops at later Braak stages (sequential collapse) [5]
Mild cognitive impairment	—	—	↓ Fewer dividing neuroblasts; 3054 ± 1149 across subjects [6]	↓ Fewer proliferating neuroblasts MCI + AD trend lower [6]	—	—
Major depressive disorder	↓ NSC density reduced [8]	↑ increased if treated; → unchanged if untreated [13]	↓ Proliferative capacity reduced [8]	→ Unchanged (early proliferative stages disrupted, not maturation) [8]	→ Unchanged [8]	—
Schizophrenia/Bipolar disorder	↓ NSC density and proliferative capacity reduced [8]	—	↓ Proliferative capacity reduced [8]	↑ Paradoxically increased (possible stalled maturation) [8]	↑ Paradoxically increased (possibly compensatory) [8]	—

↑ indicates an increase; ↓ indicates a decrease; → indicates no change; Dashes indicate the absence of available postmortem data for that marker-condition combination.

**Table 3:** Humanised environmental enrichment and potential associations with adult hippocampal neurogenesis markers and their associated health outcomes.

	Human EE Mechanism	
	Physical Activity Pathway	Spatial Complexity Pathway
Humanised environmental enrichment application	Outdoor walking [27,28,29,30,31]; stair use [34]	Urban complexity [36,37,38]; complex building layouts [39]
BDNF changes	↑ Peripheral BDNF, serum and plasma [14,27,28,29,30,31]	—
Dependent clinical outcome	—	AD [38].
Neurogenic phase potentially associated with the clinical outcome	↑ Proliferation; MDD (early proliferative stages disrupted) [8,13,14]	↑ Survival/maturation; AD/MCI (maturation stage collapse) [5,6,7]; ↑ Hippocampal *BDNF* gene expression [7]

↑ indicates an increase.

**Table 4:** Cross-species mapping of environmental enrichment mechanisms to human analogues, neurogenic markers, and clinical outcomes.

Rodents			Insufficient Approaches That Can Fail to Support Neurogenesis	Humans		
Enrichment Mechanism	Neurogenic Stage	Neurogenic Markers		Enrichment Mechanism	Clinical Outcome	Neurogenic Markers
Running wheel (voluntary physical activity)	Proliferation	Ki-67^+^, BrdU^+^ at 24 h [17,24]	Increased area or adding only one extra floor [22,34]	Outdoor walking; indoor stair use	↑ Peripheral BDNF, serum and plasma [27,28,29,30,31,47]; low depression risk in multi-storey house residents [48]	↓ Sox2^+^, ↓ Ki-67^+^/PCNA^+^ in MDD [8,13]
Complex maze (spatial complexity)	Survival and maturation	DCX^+^, PSA-NCAM^+^, NeuN^+^ [22,25,26]	Open plan layouts without points of interest [39]	Complex Zipcode [38]; complex building layouts [39]	Larger hippocampus; lower AD risk [36,38]; ↑ Hippocampal *BDNF* gene expression [7]	↓ DCX^+^, ↓ PSA-NCAM^+^, ↓ NeuN^+^ in AD and MCI [5,6,7]

↑ indicates an increase; ↓ indicates a decrease.

## Data Availability

The original contributions presented in this study are included in the article. Further inquiries can be directed to the corresponding author.
